# Efficacy, safety, and patient-reported outcome of immune checkpoint inhibitor in gynecologic cancers: A systematic review and meta-analysis of randomized controlled trials

**DOI:** 10.1371/journal.pone.0307800

**Published:** 2024-08-12

**Authors:** Fitriyadi Kusuma, Glenardi Glenardi, Ghea Mangkuliguna, Hariyono Winarto, Gatot Purwoto, Tofan Widya Utami, Tricia Dewi Anggraeni

**Affiliations:** 1 Division of Oncology Gynecology, Department of Obstetrics and Gynecology, Dr. Cipto Mangunkusumo Hospital, Greater Jakarta, Daerah Khusus Ibukota Jakarta, Indonesia; 2 School of Medicine and Health Sciences, Department of Medicine, Atma Jaya Catholic University of Indonesia, North Jakarta, Daerah Khusus Ibukota Jakarta, Indonesia; 3 Lewoleba General Hospital, Lembata Island, East Nusa Tenggara, Indonesia; Maria Sklodowska-Curie National Research Institute of Oncology Krakow, POLAND

## Abstract

Over the past decades, immune checkpoint inhibitors (ICIs) have shown dramatic efficacy in improving survival rates in multiple malignancies. Recently, gynecological cancer patients also showed to respond favorably to ICI treatment. This study aimed to evaluate the efficacy, safety, and patient-reported outcomes of ICI therapy in gynecological cancers. We conducted a systematic review and meta-analysis by retrieving literature from multiple electronic databases, such as MEDLINE, ScienceDirect, EBSCO, ProQuest, and Google Scholar. The protocol used in this study has been registered in PROSPERO (CRD42022369529). We included a total of 12 trials involving 8 therapies and 8,034 patients. ICI group demonstrated a longer OS (HR: 0.807; 95% CI: 0.719, 0.907; p = 0.000) and greater PFS improvement (HR: 0.809; 95% CI: 0.673, 0.973; p = 0.024) compared to the control group. There was no significant difference in the incidence of treatment-related adverse events [RR: 0.968; 95%CI: 0.936, 1.001; p = 0.061], but a higher incidence of immune-related adverse events (IRAEs) was observed in the ICI group (RR: 3.093; 95%CI: 1.933, 4.798; p = 0.000). Although the mean changes of QOL score from baseline was not significantly different between both groups (SMD: 0.048; 95% CI: -0.106, 0.202; p = 0.542), the time to definitive QOL deterioration was longer in the ICI group (HR: 0.508; 95% CI: 0.461, 0.560; p = 0.000). Despite having a higher incidence of IRAE, ICI was shown to improve survival rates and QOL of patients. Thus, it should be considered as a new standard of care for gynecologic cancers, especially in advanced stages.

## Introduction

Immune checkpoint inhibitors (ICIs), such as cytotoxic T lymphocyte-associated antigen 4 (CTLA-4), programmed death-1 (PD-1), and its ligand (PD-L1), represent a breakthrough in cancer treatment that successfully increases the survival rate of the patients [1, 2]. These immune checkpoints naturally exist to control immune responses in the body, and blocking them allows the host’s T-cells to regain their immune activity against cancer cells that have developed ways to evade the immune system. However, inherent characteristics of the tumor, the composition of the tumor microenvironment (TME), and deficiencies in the host’s innate and adaptive immune systems can impede the efficacy of the anti-tumor immune response and impact the host’s response to ICI therapy [1, 3].

Patients with gynecological cancers possess the potential to respond favorably to ICI treatment. Pembrolizumab was approved by the Food and Drug Administration (FDA) as a treatment for advanced cervical and endometrial cancer [2, 4–12]. Existing evidence showed conflicting results on the benefits of ICI therapy, especially in ovarian cancer [13–17], but none had directly compared the efficacy of ICI across gynecological cancers. There is also a growing body of evidence suggesting the use of ICI in the early stages of cancer or in previously untreated patients [6, 7, 15], indicating the possibility of ICI as an alternative or neoadjuvant of current existing therapy. ICI treatment can potentially lead to immune-related adverse events (IRAEs), affecting organs such as the skin, gastrointestinal tract, lungs, liver, nervous system, and endocrine system [18]. Therefore, it is necessary to understand whether the risks outweigh the benefits of ICI therapy, thus this study aims to evaluate the efficacy, safety, and patient-reported outcomes of ICI therapy in gynecological malignancies.

## Materials and methods

This meta-analysis was conducted according to the Preferred Reporting Items for Systematic Reviews and Meta-Analyses (PRISMA) criteria [19]. As this paper did not directly involve human subjects, while only using data from publicly published articles, institutional review board approval was not required. The protocol of this study has been registered in the International Prospective Register of Systematic Reviews (PROSPERO) (CRD42022369529).

### Eligibility criteria

#### Type of Study

Only randomized controlled trials (RCT) evaluating the use of ICI in gynecologic cancer were included in this study. Nonrandomized clinical studies, single-arm studies, observational studies, case series, case reports, reviews, and commentary articles were excluded. Papers with unavailable full text were also omitted.

#### Population

Subjects diagnosed with gynecologic cancer. There were no restrictions on age, race, stage, occupation, economy or social status, religion, country, underlying conditions, etc.

#### Intervention

Studies evaluating ICI for the treatment of gynecologic cancer were included in this study.

#### Comparison

Subjects treated with placebo and/or standard-of-care treatment were used as comparators.

#### Outcome

Outcomes of interest were efficacy, safety, and patient-reported outcomes of ICI for the treatment of gynecologic cancer. The efficacy assessment included overall survival (OS), progression-free survival (PFS), objective response rate (ORR), disease control rate (DCR), and duration of response (DOR). Safety assessment included the incidence of adverse events of any causes (AE) and treatment-related adverse events (TRAE) reported during treatment with ICI. Patient-reported outcome assessment included the mean changes of health-related quality of life (HRQOL) score from baseline, the proportion of patients with significant HRQOL improvement, and time to definitive HRQOL deterioration (TTD). HRQOL was assessed using the European Organization for Research and Treatment of Cancer Quality of Life Questionnaire-Core 30 (EORTC QLQ-C30) or EuroQol-5 dimension-5 level (EQ-5D) or Functional Assessment of Cancer Therapy–Ovarian (FACT-O) at baseline, during treatment, and after treatment.

### Data sources and search strategy

The systematic literature search was performed by three researchers (FK, GG, GM) using multiple electronic databases, such as PubMed/MEDLINE, ScienceDirect, EBSCO, ProQuest, and Google Scholar to identify articles published up to December 2023. The keywords used were presented in the S1 Table. After removing duplicates and irrelevant articles, three reviewers independently screened the titles and abstracts according to the eligibility criteria described above. The decision for inclusion or exclusion of articles was reached by seven investigators. Any emerging discrepancies would be resolved by consensus among the review team. A PRISMA flow chart describing the study selection process was provided (S1 Fig).

### Data extraction

Quality of Reporting of Meta-analyses and Cochrane Collaboration guidelines were used for data extraction. Seven authors (FK, GG, GM, HW, GP, TWU, TDA) independently extracted information from all included studies to ensure the quality of the data. The following data were extracted from the eligible studies: (1) study name, (2) ICI drugs and their mechanism of action, (3) study design, (4) type of cancer, (5) patient status, (6) \age of the patient, (7) treatment arm and size, (8) control arm and size, (9) dosage of ICI, and (10) median duration of follow up.

### Quality assessment

Cochrane risk of bias (RoB)2 tool was used to assess the quality of included studies based on random sequence generation, allocation concealment, blinding of participants and personnel, blinding of outcome assessment, insufficient outcome data, selective reporting of results, and other possible sources of bias [20]. The quality assessment was performed by all authors FK, GG, GM, HW, GP, TWU, and TDA independently with any discrepancies resolved by consensus among the review team.

### Statistical analysis

Hazard ratio (HR), risk ratio (RR), standardized mean differences (SMDs), and odds ratio (OR) with a confidence interval (CI) of 95% were used to determine the efficacy, safety, and patient-reported outcome of ICI for treatment of gynecologic cancer. HR was used to evaluate the effect of OS, PFS, DOR, and TTD. RR was used to evaluate the effect of ORR, DCR, AE, and TRAE. SMD was used to evaluate mean changes in HRQOL score from baseline. OR was used to evaluate the proportion of patients with significant HRQOL improvement. The effect was considered significant if the value was not equal to 1 with p < 0.05. The combined effect size was plotted using a forest plot. All the meta-analyses were performed using the random-effects model. Cochrane Q test of homogeneity and Higgins I2 were used to detect the statistical heterogeneity in studies. If heterogeneity (p >0.05 or I2 >50%) was detected, subgroup analysis was conducted to explore the possible cause of heterogeneity [21]. The publication bias was assessed visually using a funnel plot and confirmed through the Begg and Mazumdar rank correlation test and Egger test of the intercept to determine the presence of publication bias statistically [22, 23]. Duval and Tweedie’s trim-and-fill method was used to adjust the effect size if publication bias was detected [24]. Sensitivity analysis was performed to confirm the robustness of this meta-analysis by excluding studies with a high risk of bias from the analysis. All statistical tests were performed using Comprehensive Meta-Analysis software (version 3; Biostat, Englewood, NJ, USA) [25].

### Confidence in cumulative evidence

The confidence in cumulative evidence was determined using the Grading of Recommendations, Assessment, Development, and Evaluations (GRADE) framework. There are four domains assessed in the GRADE framework, namely the presence of bias, imprecision, inconsistency, and indirectness. The overall quality of evidence was shown as very low, low, moderate, and high quality.

## Results

### Search results

An initial search of electronic databases yielded 36,621 studies. After excluding 12,454 duplicate studies and 18,010 non-clinical studies, 5,797 studies were then screened. Another 5,782 studies were excluded as they were non-RCT studies, assessing other diseases, and assessing other drugs. After these rounds of exclusion, 15 studies were then assessed for eligibility for the present study. At last, 12 studies were included in our study and 3 studies were excluded due to insufficient data for extraction. The search strategy and selection methods of this study are illustrated in S1 Fig.

### Study characteristics

The majority of included studies were conducted in patients with ovarian cancer (5 studies), followed by cervical cancer (4 studies), and endometrial cancer (3 studies). Most of the included studies were conducted only on advanced gynecological cancers, whereas five other studies included all stages of cancer. This study included 6 studies using anti-PD-1 and 6 studies using anti-PD-L1 as their ICI regimen with the majority of studies using it as a combination therapy. Seven studies used placebo as a comparison, while the other seven studies used other drugs, such as pemetrexed, pegylated liposomal doxorubicin, gemcitabine, topotecan, irinotecan, or vinorelbine. In this study, 6 studies were only conducted on previously treated patients, 4 studies were only conducted on previously untreated patients, and 2 studies included all patients regardless of their treatment status. All studies showed a low risk of possible bias (S2 Fig). Characteristics of the included studies are presented in Table 1.

**Table 1:** Study characteristics.

Study	ICI drugs (mechanism of action)	Type of cancer	Patient status	Median age (years)	Treatment arm (n)	Control arm (n)	Dosage of ICI	Median duration of follow up (mo)	Median PFS (mo)	Median OS (mo)
KEYNOTE-826 trial [4, 7]	Pembrolizumab (anti-PD-1)	Cervical cancer	Persistent, Recurrent, or Metastatic Cervical Cancer (FIGO stage I-IVb). ECOG performance status ≤ 1. Number of prior lines of therapy was ≤2	51* vs 50^‡^	Pembrolizumab + paclitaxel + cisplatin or carboplatin ± bevacizumab (n = 158)	Placebo+ paclitaxel + cisplatin/ carboplatin ± bevacizumab (n = 159)	200 mg IV every 3 weeks for up to 35 cycles	22	10.4* vs 8.2^‡^ P value: <0.001	24.4* vs 16.5^‡^ P value: <0.001
EMPOWER-Cervical 1 trial [8, 9]	Cemiplimab (anti-PD-1)	Cervical cancer	Recurrent, or Metastatic Cervical Cancer (FIGO stage I-IVb) that had been treated with platinum-containing therapy. Patient have been offered or received bevacizumab and paclitaxel therapy but was rejected or discontinued due to toxicity or disease progression or logistical reasons. ECOG performance status ≤ 1. Number of prior lines of therapy was ≥1.	51* vs 50^‡^	Cemiplimab (n = 304)	Pemetrexed or topotecan or irinotecan or gemcitabine or vinorelbine (n = 304)	350 mg IV every 3 weeks	18.2	2.8* vs 2.9^‡^ P value: <0.001	12* vs 8.5^‡^ P value: <0.001
BEATcc (ENGOT-Cx10/GEICO 68-C/JGOG1084/GOG-3030) trial [5]	Atezolizumab (anti-PD-L1)	Cervical cancer	Previously untreated advanced, metastatic, or recurrent cervical cancer (FIGO stage IVb). ECOG performance status ≤ 1	51* vs 52^‡^	Atezolizumab + cisplatin/ carboplatin + bevacizumab (n = 206)	Cisplatin/ carboplatin + bevacizumab (n = 204)	1,200 mg IV every 3 weeks	32.9	13.7* vs 10.4^‡^ P value: <0.001	32.1* vs 22.8^‡^ P value: 0.005
CALLA trial [6]	Durvalumab (anti-PD-L1)	Cervical cancer	Previously untreated locally advanced cervical cancer FIGO stage IB2-IIB lymph node positive, and stage ≥III any lymph node status. ECOG performance status ≤ 1	50* vs 48^‡^	Durvalumab + external beam radiotherapy + cisplatin/ carboplatin + image-guided brachytherapy (n = 385)	Placebo + external beam radiotherapy + cisplatin/ carboplatin + image-guided brachytherapy (n = 385)	1,500 mg IV every 4 weeks	18.5* vs 18.4^‡^	NA	NA
JAVELIN Ovarian 200 trial [17]	Avelumab (anti-PD-L1)	Ovarian cancer	Platinum-resistant or platinum-refractory ovarian cancer. ECOG performance status 0–4. Number of prior lines of therapy was 1–3.	61* vs 60^†^ vs 60^‡^	Intervention group 1: avelumab alone (n = 188) Intervention group 2: avelumab + pegylated liposomal doxorubicin (n = 188)	Pegylated liposomal doxorubicin (n = 190)	10 mg/kg IV every 2 weeks	18.2* vs 18.4^†^ vs 17.4^‡^	1.9* vs 3.7^‡^ P value: >0.999 4.7^†^ vs 3.7^‡^ P value: 0.030	11.8* vs 13.1^‡^ P value: 0.830 15.7^†^ vs 13.1^‡^ P value: 0.210
JAVELIN Ovarian 100 trial [15]	Avelumab (anti-PD-L1)	Ovarian cancer	Previously untreated ovarian cancer (FIGO stage III-IV) that had undergone debulking surgery or is eligible for neoadjuvant chemotherapy. ECOG performance status ≤ 2.	59* vs 60^†^ vs 57^‡^	Intervention group 1: paclitaxel + carboplatin → avelumab (n = 332) Intervention group 2: paclitaxel + carboplatin + avelumab → avelumab (n = 331)	Paclitaxel + carboplatin → observation (n = 335)	10 mg/kg IV every 3 weeks (initial phase) and every 2 (maintenance phase)	NA	16.8* vs NE^‡^ P value: 0.990 18.1^†^ vs NE^‡^ P value: 0.790	12.6* vs 11.8^‡^ P value: 0.885 12.5^†^ vs 11.8^‡^ P value: 0.895
ATALANTE/ENGOT-ov29 trial [14]	Atezolizumab (anti-PD-L1)	Ovarian cancer	Advanced or recurrent ovarian cancer that had been treated with one platinum-containing therapy. ECOG performance status ≤ 2. Number of prior lines of therapy was ≥1.	60* vs 64^‡^	Atezolizumab+ carboplatin + gemcitabine / paclitaxel / pegylated liposomal doxorubicin + bevacizumab (n = 410)	Placebo + carboplatin + gemcitabine / paclitaxel / pegylated liposomal doxorubicin + bevacizumab (n = 204)	1,200 mg IV every 3 weeks	36.6	13.5* vs 11.3^‡^ P value: 0.041	35.5* vs 30.6^‡^ P value: NA
NINJA trial [13]	Nivolumab (anti-PD-1)	Ovarian cancer	Platinum-resistant ovarian cancer (FIGO stage I-IV). ECOG performance status ≤ 1. Number of prior lines of therapy was ≥1.	58* vs 60^‡^	Nivolumab (n = 157)	Gemcitabine or pegylated liposomal doxorubicin (n = 159)	240 mg IV every 2 weeks	NA	2.0* vs 3.8^‡^ P value: 0.002	10.1* vs 12.1^‡^ P value: 0.808
IMagyn050/GOG 3015/ENGOT-OV39 trial [16]	Atezolizumab (anti-PD-L1)	Ovarian cancer	Advanced ovarian cancer (FIGO stage III-IV) that had undergone debulking surgery or is eligible for neoadjuvant chemotherapy. ECOG performance status ≤ 2.	60* vs 59^‡^	Atezolizumab + paclitaxel + carboplatin + bevacizumab (n = 651)	Placebo + paclitaxel + carboplatin + bevacizumab (n = 650)	1,200 mg IV every 3 weeks	19.9 vs 19.8^‡^	19.5* vs 18.4^‡^ P value: 0.280	50.5* vs 46.6^‡^ P value: 0.789
NRG-GY018 trial [10]	Pembrolizumab (anti-PD-1)	Endometrial cancer	Advanced, metastatic, or recurrent endometrial cancer (FIGO stage III-IVb) that had been treated with adjuvant chemotherapy. ECOG performance status ≤ 2.	dMMR cohort: 67 vs 66^‡^ pMMR cohort: 66 vs 65^‡^	Pembrolizumab + paclitaxel + carboplatin (n = 405)	Placebo+ paclitaxel + carboplatin (n = 408)	200 mg IV every 3 weeks for up to 20 cycles	dMMR cohort: 12 pMMR cohort: 7.9	13.1* vs 8.7^‡^ P value: <0.001	NA
RUBY trial [12]	Dostarlimab (anti-PD-1)	Endometrial cancer	Advanced or recurrent endometrial cancer (FIGO stage III-IV) that was not amenable to curative treatment. ECOG performance status ≤ 1. Number of prior lines of therapy was ≤1.	64* vs 65^‡^	Dostarlimab+ paclitaxel + carboplatin (n = 245)	Placebo+ paclitaxel + carboplatin (n = 249)	200 mg IV every 3 weeks for the first six cycles, 1,000 mg IV every 6 weeks for up to 3 years	24.8	36.1* vs 18.1^‡^ P value: <0.001	71.3* vs 56.0^‡^ P value: 0.002
KEYNOTE-775 trial [11]	Pembrolizumab (anti-PD-1)	Endometrial cancer	Advanced, recurrent, or metastatic endometrial cancer that had been treated with one platinum-containing therapy. ECOG performance status ≤ 1. Number of prior lines of therapy was ≥1.	64* vs 65^‡^	Pembrolizumab + lenvatinib (n = 411)	Paclitaxel or doxorubicin (n = 416)	200 mg IV every 3 weeks for up to 35 cycles	12.2* vs 10.7^‡^	7.2* vs 3.8^‡^ P value: <0.001	18.3* vs 11.4^‡^ P value: <0.001

*, intervention group 1;

^†^, intervention group 2;

^‡^, control

Abbreviations: **ICI**: Immune checkpoint inhibitors; **PFS**: Progression-free survival; **OS**: Overall survival; **PD-1**: Programmed cell death protein 1; **PD-L1**: Programmed death ligand 1; **FIGO**: International federation of gynecology and obstetrics; **ECOG**: Eastern cooperative oncology group; **NA**: Not available; **NE**: Not estimable.

### Overall survival

The pooled data from 12 studies, involving 8,336 patients demonstrated the ICI group had a longer OS than the control group (HR: 0.807; 95% CI: 0.719, 0.907; p = 0.000; S3 Fig). However, a substantial heterogeneity (I^2^ = 60.80; p = 0.001) was observed. Therefore, subgroup analyses were done and summarized in Table 2. Subgroup analyses based on age, ECOG performance status, race, cancer stage, number of prior lines of therapy, and prior use of bevacizumab showed no significant differences in OS. Subgroup analysis demonstrated a longer OS in patients treated with anti-PD-1 drugs (HR: 0.715; 95% CI: 0.622, 0.822; p = 0.000; I^2^ = 47.54), patients with PD-L1 positive status (HR: 0.747; 95% CI: 0.637, 0.876; p = 0.000; I^2^ = 20.01), patients with cervical cancer (HR: 0.726; 95% CI: 0.646, 0.815; p = 0.000; I^2^ = 0) and endometrial cancer (HR: 0.658; 95% CI: 0.573, 0.755; p = 0.000; I^2^ = 0), previously treated patients (HR: 0.828; 95% CI: 0.719, 0.953; p = 0.009; I^2^ = 65.08), patients who treated with combination therapy (HR: 0.775; 95% CI: 0.685, 0.877; p = 0.000; I^2^ = 51.81), patients that had history of prior radiotherapy (HR: 0.731; 95% CI: 0.541, 0.988; p = 0.042; I^2^ = 3.23), and when ICI was compared with placebo as control regimen (HR: 0.768; 95% CI: 0.672, 0.877; p = 0.000; I^2^ = 35.33). The Egger’s weighted regression test (p = 0.397) and the Begg-Mazumdar Kendall’s Tahu (p = 0.620) showed no evidence of publication bias.

**Table 2:** Subgroup analyses of overall survival.

Analysis		Number of studies	HR	95% CI	P value	Heterogeneity (I^2^)
Overall effect		12	0.807	[0.719, 0.907]	0.000	60.80
Age	< 65 years	7	0.799	[0.682, 0.937]	0.006	60.92
	≥ 65 years	7	0.815	[0.683, 0.974]	0.024	30.54
Race	White	6	0.793	[0.665, 0.946]	0.010	65.81
	Other	7	0.779	[0.668, 0.909]	0.001	19.69
ECOG Score	0	7	0.769	[0.628, 0.939]	0.010	68.61
	≥ 1	7	0.816	[0.719, 0.927]	0.002	17.40
Type of cancer	Cervical cancer	4	0.726	[0.646, 0.815]	0.000	0
	Endometrial cancer	3	0.658	[0.573, 0.755]	0.000	0
	Ovarian cancer	5	0.958	[0.817, 1.123]	0.595	50.21
Cancer stage	Early and advanced stage	5	0.808	[0.672, 0.971]	0.023	74.97
	Advanced stage only	7	0.810	[0.693, 0.946]	0.008	46.42
ICI regimen	Anti-PD-1	6	0.715	[0.622, 0.822]	0.000	47.54
	Anti-PD-L1	6	0.902	[0.780, 1.043]	0.162	47.60
PD-L1 status	PD-L1 negative	5	1.020	[0.896, 1.161]	0.766	0
	PD-L1 positive	6	0.747	[0.637, 0.876]	0.000	20.01
Treatment regimen	Monotherapy	3	0.914	[0.672, 1.244]	0.567	80.12
	Combination therapy	10	0.775	[0.685, 0.877]	0.000	51.81
Treatment status	Previously untreated	3	0.937	[0.646, 1.358]	0.731	63.72
	Previously treated	7	0.828	[0.719, 0.953]	0.009	65.08
Number of prior lines of therapy	0	3	0.937	[0.646, 1.358]	0.731	63.72
	1	4	0.848	[0.610, 1.180]	0.329	79.03
	>1	4	0.865	[0.744, 1.004]	0.057	0
Prior use of bevacizumab	No	2	0.840	[0.704, 1.001]	0.052	0
	Yes	2	0.891	[0.574, 1.383]	0.606	68.95
History of prior radiotherapy	No	2	0.777	[0.408, 1.479]	0.442	92.33
	Yes	2	0.731	[0.541, 0.988]	0.042	3.23
Control regimen	Placebo	6	0.768	[0.672, 0.877]	0.000	35.33
	Non-placebo	6	0.873	[0.719, 1.060]	0.170	73.51

### Progression-free survival

The pooled data from 12 studies, involving 8,336 patients revealed a greater PFS improvement in the ICI group compared to the control group (HR: 0.809; 95% CI: 0.673, 0.973; p = 0.024; S3 Fig). However, a substantial heterogeneity (I^2^ = 90.15; p = 0.000) was observed. Therefore, subgroup analyses were done and summarized in Table 3. to explore the heterogeneity. Subgroup analyses based on age, race, PD-L1 status, treatment status, number of prior lines of therapy, prior use of bevacizumab, and history of prior radiotherapy showed no significant differences in PFS. Subgroup analysis showed significant improvement in PFS for patients with ECOG score of 0 (HR: 0.774; 95% CI: 0.608, 0.985; p = 0.037; I^2^ = 82.31), patients treated with anti-PD-1 drugs (HR: 0.654; 95% CI: 0.494, 0.865; p = 0.003; I^2^ = 91.13), patients with cervical cancer (HR: 0.708; 95% CI: 0.625, 0.800; p = 0.000; I^2^ = 27.30) and endometrial cancer (HR: 0.528; 95% CI: 0.429, 0.653; p = 0.000; I^2^ = 64.12), patients with advanced stage cancer (HR: 0.758; 95% CI: 0.612, 0.938; p = 0.011; I^2^ = 94.22), patients who treated with combination therapy (HR: 0.729; 95% CI: 0.616, 0.864; p = 0.000; I^2^ = 84.75), and when ICI was compared with non-placebo as control regimen (HR: 0.970; 95% CI: 0.713, 1.319; p = 0.000; I^2^ = 81.97). The Egger’s weighted regression test (p = 0.773) and the Begg-Mazumdar Kendall’s Tahu (p = 1.000) showed no evidence of publication bias.

**Table 3:** Subgroup analyses of progression-free survival.

Analysis		Number of studies	HR	95% CI	P value	Heterogeneity (I^2^)
Overall effect		12	0.809	[0.673, 0.973]	0.024	90.15
Age	< 65 years	9	0.773	[0.626, 0.955]	0.017	82.93
	≥ 65 years	9	0.748	[0.583, 0.961]	0.023	77.08
Race	White	8	0.776	[0.608, 0.989]	0.040	87.60
	Other	9	0.752	[0.642, 0.881]	0.000	34.60
ECOG Score	0	7	0.774	[0.608, 0.985]	0.037	82.31
	≥ 1	7	0.800	[0.601, 1.065]	0.126	83.89
Type of cancer	Cervical cancer	4	0.708	[0.625, 0.800]	0.000	27.30
	Endometrial cancer	3	0.528	[0.429, 0.653]	0.000	64.12
	Ovarian cancer	5	1.127	[0.898, 1.414]	0.303	85.50
Cancer stage	Early and advanced stage	5	0.896	[0.633, 1.269]	0.537	85.58
	Advanced stage only	7	0.758	[0.612, 0.938]	0.011	94.22
ICI regimen	Anti-PD-1	6	0.654	[0.494, 0.865]	0.003	91.13
	Anti-PD-L1	6	0.971	[0.787, 1.198]	0.781	84.84
PD-L1 status	PD-L1 negative	6	1.035	[0.878, 1.220]	0.680	39.28
	PD-L1 positive	6	0.813	[0.653, 1.013]	0.065	69.62
Treatment regimen	Monotherapy	3	1.229	[0.720, 2.099]	0.449	94.58
	Combination therapy	10	0.729	[0.616, 0.864]	0.000	84.75
Treatment status	Previously untreated	3	0.950	[0.661, 1.365]	0.083	85.68
	Previously treated	7	0.793	[0.611, 1.031]	0.779	92.76
Number of prior lines of therapy	0	3	0.949	[0.661, 1.364]	0.779	85.63
	1	3	0.892	[0.548, 1.451]	0.645	92.42
	>1	3	0.823	[0.582, 1.204]	0.315	77.07
Prior use of bevacizumab	No	2	0.957	[0.561, 1.362]	0.872	90.02
	Yes	3	0.892	[0.694, 1.146]	0.37	66
History of prior radiotherapy	No	3	0.623	[0.459, 0.847]	0.003	72.08
	Yes	3	0.622	[0.543, 0.713]	0.000	3.86
Control regimen	Placebo	6	0.672	[0.550, 0.822]	0.847	93.14
	Non-placebo	6	0.970	[0.713, 1.319]	0.000	81.97

### Objective response, disease control, and duration of response

The clinical response was evaluated using ORR, DCR, and DOR. A total of 11 studies demonstrated that the ICI group had a significantly higher ORR than the control group (RR: 1.186; 95% CI: 1.065, 1.321; p = 0.002; S3 Fig). Subgroup analysis (Table 4) was done and demonstrated a higher ORR in patients treated with anti-PD-1 drugs (HR: 1.448; 95% CI: 1.036, 2.023; p = 0.030; I^2^ = 89.93), patients with cervical cancer (HR: 1.244; 95% CI: 1.041, 1.486; p = 0.016; I^2^ = 86.77), patients with early and advanced stage cancer (HR: 1.628; 95% CI: 1.078, 2.459; p = 0.021; I^2^ = 83.01), previously treated patients (HR: 1.392; 95% CI: 1.043, 1.856; p = 0.025; I^2^ = 72.71), patients treated with combination therapy (HR: 1.164; 95% CI: 1.051, 1.290; p = 0.004; I^2^ = 82.74), and when ICI was compared with non-placebo as control regimen (HR: 1.408; 95% CI: 1.072, 1.850; p = 0.014; I^2^ = 83.94). In contrast, there were no significant DCR differences among these two groups (RR: 1.021; 95% CI: 0.930, 1.120; p = 0.667; S3 Fig). However, subgroup analysis (Table 4) showed a higher DCR in patients with cervical cancer (HR: 1.051; 95% CI: 1.009, 1.095; p = 0.018; I^2^ = 0), patients treated with combination therapy (HR: 1.096; 95% CI: 1.006, 1.195; p = 0.036; I^2^ = 88.71), and when ICI was compared with placebo as control regimen (HR: 1.044; 95% CI: 1.044, 1.086; p = 0.030; I^2^ = 0). However, there was a lower DCR in previously treated patients (HR: 0.951; 95% CI: 0.708, 1.276; p = 0.000; I^2^ = 93.21). The DOR was longer in the ICI group than in the control group (HR: 0.581; 95% CI: 0.440, 0.767; p = 0.000; S3 Fig), though there was a moderate heterogeneity observed (I^2^ = 57.22; p = 0.071). Subgroup analysis (Table 4) was done and demonstrated a longer DOR in patients with cervical cancer (HR: 0.643; 95% CI: 0.529, 0.782; p = 0.000; I^2^ = 0), patients with early and advanced stage cancer (HR: 0.572; 95% CI: 0.470, 0.697; p = 0.000; I^2^ = 0), and when ICI was compared with placebo as control regimen (HR: 0.615; 95% CI: 0.477, 0.794; p = 0.000; I^2^ = 33.95). The Egger’s weighted regression test and the Begg-Mazumdar Kendall’s Tahu showed no evidence of publication bias for these meta-analyses.

**Table 4:** Subgroup analyses of objective response rate, disease control rate and duration of response.

Analysis		Number of studies	Effect size	95% CI	P value	Heterogeneity (I^2^)
Meta-analysis: objective response rate (ORR)						
Overall effect		11	1.186	[1.065, 1.321]	0.002	83.23
Type of cancer	Cervical cancer	4	1.244	[1.041, 1.486]	0.016	86.77
	Endometrial cancer	2	1.555	[0.730, 3.311]	0.252	95.90
	Ovarian cancer	5	1.042	[0.917, 1.184]	0.527	58.05
Cancer stage	Early and advanced stage	5	1.628	[1.078, 2.459]	0.021	83.01
	Advanced stage only	6	1.052	[1.000, 1.107]	0.050	35.07
ICI regimen	Anti-PD-1	5	1.448	[1.036, 2.023]	0.030	89.93
	Anti-PD-L1	6	1.062	[0.985, 1.145]	0.115	59.23
Treatment regimen	Monotherapy	3	1.151	[0.409, 3.241]	0.790	84.19
	Combination therapy	9	1.164	[1.051, 1.290]	0.004	82.74
Treatment status	Previously untreated	3	1.087	[0.995, 1.188]	0.065	89.77
	Previously treated	6	1.392	[1.043, 1.856]	0.025	72.71
Control regimen	Placebo	5	1.060	[0.962, 1.145]	0.135	68.94
	Non-placebo	6	1.408	[1.072, 1.850]	0.014	83.94
Meta-analysis: disease control rate (DCR)						
Overall effect		8	1.021	[0.930, 1.120]	0.667	86.69
Type of cancer	Cervical cancer	3	1.051	[1.009, 1.095]	0.018	0
	Endometrial cancer	2	1.205	[0.853, 1.703]	0.289	95.99
	Ovarian cancer	3	0.898	[0.751, 1.075]	0.242	84.32
Cancer stage	Early and advanced stage	5	0.983	[0.808, 1.197]	0.868	91.91
	Advanced stage only	3	1.029	[0.988, 1.071]	0.168	0
ICI regimen	Anti-PD-1	5	1.030	[0.866, 1226]	0.738	91.95
	Anti-PD-L1	3	1.005	[0.915, 1.104]	0.919	69.71
Treatment regimen	Monotherapy	3	0.759	[0.519, 1.110]	0.155	84.23
	Combination therapy	6	1.096	[1.006, 1.195]	0.036	88.71
Treatment status	Previously untreated	2	1.033	[0.988, 1.080]	0.156	0
	Previously treated	4	0.951	[0.708, 1.276]	0.000	93.21
Control regimen	Placebo	3	1.044	[1.044, 1.086]	0.030	0
	Non-placebo	5	0.984	[0.826, 1.172]	0.857	90.78
Meta-analysis: duration of response (DOR)						
Overall effect		4	0.581	[0.440, 0.767]	0.000	57.22
Type of cancer	Cervical cancer	2	0.643	[0.529, 0.782]	0.000	0
	Endometrial cancer	2	0.238	[0.033, 1.694]	0.152	79.13
Cancer stage	Early and advanced stage	2	0.572	[0.470, 0.697]	0.000	0
	Advanced stage only	2	0.265	[0.029, 2.461]	0.243	83.58
Control regimen	Placebo	2	0.615	[0.477, 0.794]	0.000	33.95
	Non-placebo	2	0.249	[0.031, 1.972]	0.188	81.22

### Safety

There was no significant difference in the probability of adverse events of any cause (AE) [RR: 1.000; 95%CI: 0.988, 1.012; p = 0.972; I^2^ = 82.62; S4 Fig] and treatment-related adverse events (TRAE) [RR: 0.968; 95%CI: 0.936, 1.001; p = 0.061; I^2^ = 91.74; S4 Fig]. Moreover, there was also no significant difference in the probability of grade 3–5 AE (RR: 1.024; 95%CI: 0.940, 1.116; p = 0.588; I^2^ = 88.92) and grade 3–5 TRAE (RR: 0.869; 95%CI: 0.738, 1.023; p = 0.091; I^2^ = 92.66). The probability of immune-related AE was significantly higher in the ICI group than in the control group (RR: 3.093; 95%CI: 1.933, 4.798; p = 0.000; I^2^ = 94.74). On the contrary, there were no significant differences in the immune-related TRAE in the ICI group than in the control group (RR: 1.725; 95%CI: 0.882, 3.373; p = 0.111; I^2^ = 91.87). However, the probability of TRAE-related discontinuation was significantly higher in the ICI group than in the control group (RR: 1.711; 95%CI: 1.224, 2.390; p = 0.002; I^2^ = 79.42). The Egger’s weighted regression test and the Begg-Mazumdar Kendall’s Tahu showed no evidence of publication bias for these meta-analyses. In addition, to explore substantial heterogeneity in these meta-analyses. subgroup analyses were also done and summarized in Table 5.

**Table 5:** Subgroup analyses of adverse events of any cause and treatment-related adverse events.

Analysis		Number of studies	Effect size	95% CI	P value	Heterogeneity (I^2^)
Meta-analysis: adverse events of any cause (AE)						
Overall effect		10	1.000	[0.988, 1.012]	0.972	82.62
Type of cancer	Cervical cancer	4	1.000	[0.991, 1.009]	0.947	0
	Endometrial cancer	2	1.002	[0.994, 1.010]	0.659	0
	Ovarian cancer	4	0.996	[0.952, 1.020]	0.414	92.49
Cancer stage	Early and advanced stage	5	0.973	[0.944, 1.004]	0.090	91.07
	Advanced stage only	5	1.008	[0.997, 1.018]	0.148	51.90
ICI regimen	Anti-PD-1	5	0.971	[0.940, 1.002]	0.070	90.88
	Anti-PD-L1	5	1.008	[0.998, 1.019]	0.117	54.86
Treatment regimen	Monotherapy	3	0.856	[0.725, 1.011]	0.113	31.27
	Combination therapy	8	1.005	[0.999, 1.010]	0.067	95.62
Treatment status	Previously untreated	3	1.001	[0.992, 1.010]	0.796	87.61
	Previously treated	6	0.967	[0.969, 1.006]	0.191	62.45
Control regimen	Placebo	4	1.001	[0.997, 1.005]	0.524	0
	Non-placebo	6	0.989	[0.962, 1.016]	0.415	89.66
Meta-analysis: treatment-related adverse events (TRAE)						
Overall effect		9	0.968	[0.936, 1.001]	0.061	91.74
Type of cancer	Cervical cancer	3	0.911	[0.794, 1.044]	0.181	95.10
	Endometrial cancer	2	1.013	[0.969, 1.059]	0.572	81.47
	Ovarian cancer	4	0.952	[0.900, 1.008]	0.090	93.19
Cancer stage	Early and advanced stage	5	0.878	[0.790, 0.976]	0.016	95.48
	Advanced stage only	4	1.007	[0.990, 1.025]	0.424	61.45
ICI regimen	Anti-PD-1	5	0.883	[0.811, 0.961]	0.004	95.88
	Anti-PD-L1	4	1.011	[0.980, 1.042]	0.487	78.75
Treatment regimen	Monotherapy	3	0.720	[0.607, 0.854]	0.000	84.894
	Combination therapy	7	1.013	[0.997, 1.030]	0.110	65.713
Treatment status	Previously untreated	2	1.025	[1.006, 1.045]	0.011	0
	Previously treated	5	0.883	[0.810, 0.963]	0.005	95.47
Control regimen	Placebo	4	0.995	[0.987, 1.003]	0.198	0
	Non-placebo	5	0.907	[0.836, 0.984]	0.019	94.82
Meta-analysis: AE grade 3–5						
Overall effect		12	1.024	[0.940, 1.116]	0.588	89.92
Type of cancer	Cervical cancer	4	1.023	[0.932, 1.122]	0.632	60.48
	Endometrial cancer	3	1.220	[1.155, 1.290]	0.000	0
	Ovarian cancer	5	0.909	[0.783, 1.055]	0.211	92.397
Cancer stage	Early and advanced stage	5	0.843	[0.672, 1.058]	0.140	94.53
	Advanced stage only	7	1.081	[1.008, 1.159]	0.028	73.71
ICI regimen	Anti-PD-1	6	0.964	[0.796, 1.168]	0.708	93.07
	Anti-PD-L1	6	1.030	[0.965, 1.01]	0.373	69.68
Treatment regimen	Monotherapy	3	0.517	[0.277, 0.966]	0.038	94.41
	Combination therapy	10	1.102	[1.035, 1.175]	0.003	78.77
Treatment status	Previously untreated	3	1.088	[1.028, 1.151]	0.003	0
	Previously treated	7	0.946	[0.818, 1.094]	0.456	93.19
Control regimen	Placebo	6	1.078	[0.993, 1.170]	0.074	77.59
	Non-placebo	6	0.924	[0.784, 1.089]	0.343	92.46
Meta-analysis: TRAE grade 3–5						
Overall effect		10	0.869	[0.738, 1.023]	0.091	92.66
Type of cancer	Cervical cancer	3	0.745	[0.469, 1.184]	0.213	95.23
	Endometrial cancer	2	1.218	[1.021, 1.454]	0.028	66.97
	Ovarian cancer	5	0.816	[0.640, 1.039]	0.099	92.78
Cancer stage	Early and advanced stage	5	0.651	[0.426, 0.994]	0.047	96.52
	Advanced stage only	5	1.060	[0.997, 1.127]	0.062	21.07
ICI regimen	Anti-PD-1	5	0.662	[0.435, 1.008]	0.054	96.84
	Anti-PD-L1	5	1.013	[0.899, 1.141]	0.837	73.89
Treatment regimen	Monotherapy	3	0.318	[0.180, 0.562]	0.000	84.81
	Combination therapy	8	1.099	[1.019, 1.185]	0.014	64.43
Treatment status	Previously untreated	2	1.025	[0.928, 1.132]	0.630	32.09
	Previously treated	6	0.705	[0.512, 0.971]	0.033	95.88
Control regimen	Placebo	5	1.080	[1.025, 1.137]	0.004	0
	Non-placebo	5	0.702	[0.497, 0.992]	0.045	95.90
Meta-analysis: immune-related AE						
Overall effect		7	3.093	[1.993, 4.798]	0.000	94.74
Type of cancer	Cervical cancer	1	1.615	[1.296, 2.013]	0.000	0
	Endometrial cancer	3	2.927	[1.127, 7.600]	0.027	97.34
	Ovarian cancer	3	6.685	[2.089, 21.387]	0.001	90.19
Cancer stage	Early and advanced stage	2	7.356	[2.293, 23.598]	0.001	90.17
	Advanced stage only	5	1.629	[1.310, 2.026]	0.000	75.32
ICI regimen	Anti-PD-1	3	2.927	[1.127, 7.600]	0.027	97.34
	Anti-PD-L1	4	2.895	[1.745, 4.804]	0.000	87.96
Treatment regimen	Monotherapy	1	2.958	[1.371, 6.383]	0.006	95.26
	Combination therapy	7	3.114	[1.957, 4.955]	0.000	0
Treatment status	Previously untreated	2	26.158	[0.693, 987.802]	0.078	89.92
	Previously treated	4	3.209	[1.531, 6.728]	0.002	96.67
Control regimen	Placebo	4	1.464	[1.362, 1.573]	0.000	0
	Non-placebo	3	13.548	[4.450, 41.244]	0.000	85.17
Meta-analysis: immune-related TRAE						
Overall effect		4	1.725	[0.882, 3.373]	0.111	91.87
Treatment regimen	Monotherapy	2	0.906	[0.734, 1.118]	0.356	0
	Combination therapy	2	2.336	[1.864, 2.927]	0.000	0
Control regimen	Placebo	2	2.336	[1.864, 2.927]	0.000	0
	Non-placebo	2	0.906	[0.734, 1.118]	0.356	0
Meta-analysis: TRAE-related discontinuation						
Overall effect		10	1.711	[1.224, 2.390]	0.002	79.42
Type of cancer	Cervical cancer	3	1.697	[1.159, 2.485]	0.007	0
	Endometrial cancer	2	3.063	[1.188, 7.897]	0.021	90.10
	Ovarian cancer	5	1.411	[0.951, 2.094]	0.087	74.94
Cancer stage	Early and advanced stage	5	1.355	[0.646, 2.845]	0.422	88.72
	Advanced stage only	5	1.970	[1.524, 2.546]	0.000	43.83
ICI regimen	Anti-PD-1	5	1.842	[0.965, 3.516]	0.064	86.62
	Anti-PD-L1	5	1.619	[1.121, 2.337]	0.010	68.93
Treatment regimen	Monotherapy	3	1.008	[0.621, 1.636]	0.975	18.85
	Combination therapy	8	1.983	[1.384, 2.840]	0.000	80.21
Treatment status	Previously untreated	2	2.145	[1.535, 2.996]	0.000	0
	Previously treated	6	1.495	[0.850, 2.628]	0.162	88.23
Control regimen	Placebo	5	1.846	[1.384, 2.460]	0.000	47.67
	Non-placebo	5	1.524	[0.831, 2.793]	0.173	86.42

### Patients-reported outcomes

The pooled data from 6 studies revealed that mean changes in QOL score from baseline were not significantly different between both groups (SMD: 0.048; 95% CI: -0.106. 0.202; p = 0.542; I^2^ = 40.82; S5 Fig). Subgroup analysis (Table 6) showed a higher mean change of QOL score from baseline in patients with endometrial cancer (SMD: 0.306; 95% CI: 0.040. 0.572; p = 0.024; I^2^ = 0). However, there was a lower mean change of QOL score from baseline in patients with ovarian cancer (SMD: -0.177; 95% CI: -0.353. -0.002; p = 0.048; I^2^ = 0). Moreover, a meta-analysis of 4 studies also showed that the proportion of patients with significant improvement of QOL was not significantly different between both groups (OR: 1.106; 95% CI: 0.961. 1.272; p = 0.159; I^2^ = 11.48; S5 Fig). However, subgroup analysis (Table 6) showed a higher proportion of QOL improvement in patients treated with anti-PD-1 and patients with cervical cancer (OR: 1.498; 95% CI: 1.248, 1.799; p = 0.000; I^2^ = 0). Time to definitive QOL deterioration was longer in the ICI group than in the control group (HR: 0.508; 95% CI: 0.461, 0.560; p = 0.000). The Egger’s weighted regression test and the Begg-Mazumdar Kendall’s Tahu showed no evidence of publication bias for these meta-analyses.

**Table 6:** Subgroup analyses of patient-reported outcome.

Analysis		Number of studies	Effect size	95% CI	P value	Heterogeneity (I^2^)
Meta-analysis: mean changes of quality of life (QOL) score from baseline						
Overall effect		6	0.048	[-0.106, 0.202]	0.542	40.82
QOL domain	Global health status	6	0.058	[-0.124, 0.240]	0.535	47.49
	Functional domain	2	-0.104	[-0.568, 0.359]	0.659	49.90
	Symptom domain	2	0.222	[-0.225, 0.669]	0.329	12.02
Type of cancer	Cervical cancer	2	0.028	[-0.204, 0.259]	0.815	42.95
	Endometrial cancer	2	0.306	[0.040,0.572]	0.024	0
	Ovarian cancer	2	-0.177	[-0.353, -0.002]	0.048	0
Treatment regimen	Monotherapy	2	-0.106	[-0.469, 0.256]	0.566	29.24
	Combination therapy	5	0.085	[-0.077, 0.247]	0.304	34.43
Control regimen	Placebo	3	0.064	[-0.164, 0.292]	0.582	42.09
	Non-placebo	3	0.024	[-0.182, 0.229]	0.822	34.83
Meta-analysis: proportion of QOL improvement						
Overall effect		4	1.106	[0.961, 1.272]	0.159	11.48
QOL domain	Global health status	4	1.180	[0.859, 1.621]	0.308	55.91
	Functional domain	3	1.067	[0.874, 1.304]	0.523	0
	Symptom domain	2	0.920	[0.693, 1.220]	0.562	0
Type of cancer	Cervical cancer	2	1.498	[1.248, 1.799]	0.000	0
	Ovarian cancer	2	0.870	[0.735, 1.030]	0.107	0
ICI regimen	Anti-PD-1	2	1.498	[1.248, 1.799]	0.000	0
	Anti-PD-L1	2	0.870	[0.735, 1.030]	0.107	0
Treatment regimen	Monotherapy	2	1.337	[0.943, 1.897]	0.103	0
	Combination therapy	3	1.035	[0.849, 1.262]	0.732	53.22
Control regimen	Placebo	3	1.071	[0.879, 1.306]	0.496	51.53
	Non-placebo	2	1.126	[0.829, 1.528]	0.447	0
Meta-analysis: time to definitive QOL deterioration (TTD)						
Overall effect		3	0.508	[0.461, 0.560]	0.000	77.62
QOL domain	Global health status	3	0.605	[0.469, 0.780]	0.000	80.44
	Functional domain	3	0.576	[0.491, 0.677]	0.000	72.49
	Symptom domain	2	0.465	[0.410, 0.528]	0.000	75.92

### Confidence in cumulative evidence

According to the Cochrane risk of bias tool (ROB-2), all included studies were judged to have a low risk of bias, thus plausible bias was unlikely to alter the outcomes. There was also no indication of indirectness in all the outcomes of this study. In contrast, the inconsistency was serious in almost all outcomes as there was a substantial heterogeneity observed among the included studies. However, by conducting subgroup analyses, we were able to reduce the heterogeneity of each outcome and address this issue. There were also some imprecision observed especially when assessing the incidence of IRAE, immune-related TRAE, TRAE-related discontinuation, and the impact of ICIs on QOL improvement because the majority of the included studies in these outcomes had wide confidence intervals. Moreover, in the outcomes with more than 10 included studies, no publication bias was found. Overall, we had moderate quality of evidence for all the outcomes, except the outcomes for incidence of IRAE, immune-related TRAE, and TRAE-related discontinuation which were considered as low quality of evidence. GRADE evidence profile is generated as shown in S2 Table.

## Discussion

In terms of efficacy, there was a marked improvement in the clinical outcomes in the ICI group compared to control. Pooled data showed higher OS, PFS, and ORR among patients treated with ICI. The effects were mainly observed in cervical cancer, treatment with anti-PD-1 drugs, use of combination therapy, and advanced cancers. According to a model proposed by Yarchoan et al, cervical cancer had a higher tumor mutational burden compared to endometrial and ovarian cancers, suggesting that there are more neoantigens present in the tumor cells and microenvironment that leads to increased response to checkpoint inhibition. This hypothesis is grounded in the rationale that a heightened presence of neoantigens improves the detection of tumor cells by the immune system [26, 27].

Immunotherapy with anti-PD-1 seems to show superior OS, PFS, and ORR compared to anti-PD-L1 probably as a result of the simultaneous inhibition of PD-1 binding to PD-L1 and PD-L2. On the contrary, anti-PD-L1 specifically interacts with PD-1, hence only blocks the binding of PD-1 to PD-L1 and does not affect the interaction between PD-1 and PD-L2. This process may lead to the inhibition of T-cell activation and further enhance the immunologic escape of the tumor cells. Anti-PD-1 and anti-PD-L1 therapy may have similar efficacy in cases of patients having high expression of PD-L1. However, anti-PD-L1 treatment may be insufficient for patients with low-to-none PD-L1 expression or high PD-L2 expression [28, 29].

The benefits of immunotherapy with ICI are also enhanced as part of the combination regimen. This strategy involves the synergistic effect of combination treatments to generate an anti-tumor response, prevent the immunologic escape of the tumor cells, and reduce the adverse events that arise during the treatment. The combinations are designed based on the hallmark mechanisms of cancer immunotherapy, which include immunogenic cell death, enhanced antigen-presenting cells, activation of primed T cells and reversion of exhaustion, increased lymphocyte infiltration, and depletion of immunosuppressive cells [30].

It is possible that patients who responded well to ICIs are more likely to experience autoimmune toxicities [31], however, this meta-analysis showed that the safety profile of the treatment was comparable between the two groups. The occurrence of immune-related adverse events (IRAEs) is in accordance with the action of the checkpoint blockade and may be related to the antitumor responses. IRAEs mostly occur during the first three months of the treatment and frequently involve organs with significant exposure to the environment (skin, lungs, gastrointestinal tract) or those known with pre-existing or smoldering autoimmunity (thyroid, joints). Evidence suggests that patients who developed IRAEs had favorable OS, PFS, ORR, and DCR compared to those who experienced minimal toxicity [18, 31]. As shown in this meta-analysis, the occurrence of IRAEs was significantly higher in the ICI group, suggesting the potential link between IRAEs and antitumor immunity. Although we did not observe significant changes in QoL between the two groups, immunotherapy with ICI can mitigate significant QoL deterioration, as seen in the longer TTD in the ICI group. There is also a trend of superior clinical benefits after ICI therapy coupled with favorable patient-reported outcomes.

Moreover, we acknowledge some limitations in this study. The included studies in this meta-analysis are heterogeneous, thus it can be difficult to conduct a meaningful comparison. However, we mitigate this issue by using a random effects model and conducting subgroup analyses. In addition, due to the limited of studies available, our included studies were dominated by advanced-stage cancer, previously treated patients, and the use of ICI as a combination therapy, which could impact the generalizability of this study. Finally, there were some studies that were not included in our analysis because they had not been published at the time of our literature search. Nevertheless, despite these limitations, this study remains the first and most comprehensive meta-analysis addressing the use of ICI in gynecologic cancer.

## Conclusions

This meta-analysis suggested the favorable effect of ICI on gynecological cancer, especially when compared to those who received a placebo. Although the safety profile was remarkable, there is a trend of increasing IRAEs in the ICI group, highlighting the possibility of IRAE and antitumor response that generate markedly improved clinical outcomes.

## Supporting information

S1 Table Search keywords. (DOCX)

S2 Table GRADE evidence profile. (DOCX)

S1 Fig PRISMA flowchart. The diagram provided a summary of the search strategy and selection process used to include eligible articles for this meta-analysis.
(DOCX)

S2 Fig Quality assessment. Cochrane risk of bias tool (ROB-2) was used to evaluate the risk of bias.
(DOCX)

S3 Fig Overall survival, progression-free survival, objective response rate, disease control rate, and duration of response of ICI for gynecologic cancer. (Left) Forest plot. The horizontal line indicates 95% CI of a study. The square represents the result of each individual study. The size of the square varies according to the weight of a particular study. The diamond at the bottom of the plot represents the pooled analysis of all included studies. The outer edges of the diamond indicates the CIs. CI, confidence interval. (Right) Funnel plot. Asymmetrical plot indicated that publication bias was present.
(DOCX)

S4 Fig Adverse events of any causes and treatment-related adverse events of ICI for gynecologic cancer. (Left) Forest plot. The horizontal line indicates 95% CI of a study. The square represents the result of each individual study. The size of the square varies according to the weight of a particular study. The diamond at the bottom of the plot represents the pooled analysis of all included studies. The outer edges of the diamond indicates the CIs. CI, confidence interval. (Right) Funnel plot. Asymmetrical plot indicated that publication bias was present.
(DOCX)

S5 Fig Patient-reported outcomes of ICI for gynecologic cancer. (Left) Forest plot. The horizontal line indicates 95% CI of a study. The square represents the result of each individual study. The size of the square varies according to the weight of a particular study. The diamond at the bottom of the plot represents the pooled analysis of all included studies. The outer edges of the diamond indicates the CIs. CI, confidence interval. (Top right) Funnel plot. Asymmetrical plot indicated that publication bias was present.
(DOCX)

S1 Checklist (DOCX)
