# Correction: Efficacy, safety, and patient-reported outcome of immune checkpoint inhibitor in gynecologic cancers: A systematic review and meta-analysis of randomized controlled trials

**DOI:** 10.1371/journal.pone.0316871

**Published:** 2024-12-30

**Authors:** Fitriyadi Kusuma, Glenardi Glenardi, Ghea Mangkuliguna, Hariyono Winarto, Gatot Purwoto, Tofan Widya Utami, Tricia Dewi Anggraeni

There is an error in affiliation 1 for authors Fitriyadi Kusuma, Glenardi Glenardi, Hariyono Winarto, Gatot Purwoto, Tofan Widya Utami and Tricia Dewi Anggraeni. The correct affiliation 1 is: Faculty of Medicine Universitas Indonesia, Department of Obstetrics and Gynecology, Division of Oncology Gynecology, Dr. Cipto Mangunkusumo Hospital, Greater Jakarta, DKI Jakarta, Indonesia.
