# Supplementary material for: Efficacy, safety, and patient-reported outcome of immune checkpoint inhibitor in gynecologic cancers: A systematic review and meta-analysis of randomized controlled trials
Source: PLoS One. 2024 Aug 12;19(8):e0307800. doi: 10.1371/journal.pone.0307800 (PMC11318932; doi:10.1371/journal.pone.0307800)
Supplement: S1 Table — (DOCX) [file pone.0307800.s001.docx]

**Table S1.** Search Keywords

| **Databases** | **Keywords** |
| --- | --- |
| PubMed | (Cervical Cancer[Title/Abstract]) OR (Ovarian Cancer[Title/Abstract]) OR (Endometrial Cancer[Title/Abstract]) OR (Vaginal Cancer[Title/Abstract]) OR (Vulvar Cancer[Title/Abstract]) AND (Immune Checkpoint Inhibitor[Title/Abstract]) OR (ICIs[Title/Abstract]) OR (PD-1 inhibitor[Title/Abstract]) OR (PD-L1 inhibitor[Title/Abstract]) OR (Anti-PD-1[Title/Abstract]) OR (Anti-PD-L1[Title/Abstract]) OR (CTLA-4 Inhibitor[Title/Abstract]) OR (Anti-CTLA-4[Title/Abstract]) OR (Atezolizumab[Title/Abstract]) OR (Avelumab[Title/Abstract]) OR (Durvalumab[Title/Abstract]) OR (Nivolumab[Title/Abstract]) OR (Pembrolizumab[Title/Abstract]) OR (Cemiplimab[Title/Abstract]) OR (Camrelizumab[Title/Abstract]) OR (Balstilimab[Title/Abstract]) OR (Sintilimab[Title/Abstract]) OR (Ipilimumab[Title/Abstract]) OR (zalifrelimab[Title/Abstract]) |
| ScienceDirect, EBSCO, ProQuest | "Cervical Cancer" OR "Ovarian Cancer" OR "Endometrial Cancer" OR "Vaginal Cancer" OR "Vulvar Cancer" AND "Immune Checkpoint Inhibitor" OR "ICIs" OR "PD-1 inhibitor" OR "PD-L1 inhibitor" OR "Anti-PD-1" OR "Anti-PD-L1" OR "CTLA-4 Inhibitor" OR "Anti-CTLA-4" OR "Atezolizumab" OR "Avelumab" OR "Durvalumab" OR "Nivolumab" OR "Pembrolizumab" OR "Cemiplimab" OR "Camrelizumab" OR "Balstilimab" OR "Sintilimab" OR "Ipilimumab" OR "zalifrelimab" |
