# Supplementary material for: Efficacy, safety, and patient-reported outcome of immune checkpoint inhibitor in gynecologic cancers: A systematic review and meta-analysis of randomized controlled trials
Source: PLoS One. 2024 Aug 12;19(8):e0307800. doi: 10.1371/journal.pone.0307800 (PMC11318932; doi:10.1371/journal.pone.0307800)
Supplement: S2 Table — (DOCX) [file pone.0307800.s002.docx]

**Table S2.** GRADE Evidence Profile

| Immune Checkpoint Inhibitor (ICI) Compared to Control for the Treatment of Gynecologic Cancers | | | | | | | | | | |
| --- | --- | --- | --- | --- | --- | --- | --- | --- | --- | --- |
| Outcome | No. of  Participants (Studies) | Quality Assessment | | | | | | Summary of Findings | | |
|  |  | Risk of Bias | Inconsistency | Indirectness | Imprecision | Publication Bias | Overall Quality of Evidence | Study Event Rates | | Relative  Effect  (95% CI) |
|  |  |  |  |  |  |  |  | ICI | Control |  |
| Overall survival | 8,336 (12 RCTs) | Not serious | Serious^1^ | Not serious | Not serious | Not serious | ⊕⊕⊕〇 MODERATE | - | - | **HR 0.807** (0.719 to 0.907) |
| Progression-free survival | 8,336 (12 RCTs) | Not serious | Serious^1^ | Not serious | Not serious | Not serious | ⊕⊕⊕〇 MODERATE | - | - | **HR 0.809** (0.673 to 0.973) |
| Objective response rate | 6,627 (11 RCTs) | Not serious | Serious^1^ | Not serious | Not serious | Not serious | ⊕⊕⊕〇 MODERATE | 1,782 /3,678 | 1,308 /2,949 | **RR 1.186** (1.065 to 1.321) |
| Disease control rate | 5,113 (8 RCTs) | Not serious | Serious^1^ | Not serious | Not serious | N/A^2^ | ⊕⊕⊕〇 MODERATE | 1,976 /2,811 | 2,302 /5,113 | **RR 1.021** (0.930 to 1.120) |
| Duration of response | 2,348 (4 RCTs) | Not serious | Serious^1^ | Not serious | Not serious | N/A^2^ | ⊕⊕⊕〇 MODERATE | - | - | **HR 0.581** (0.440 to 0.767) |
| Incidence of AE | 7,570 (10 RCTs) | Not serious | Serious^1^ | Not serious | Not serious | Not serious | ⊕⊕⊕〇 MODERATE | 3,914 /4,055 | 3,437 /3,515 | **RR 1.000** (0.988 to 1.012) |
| Incidence of AE grade 3-5 | 8,175 (12 RCTs) | Not serious | Serious^1^ | Not serious | Not serious | Not serious | ⊕⊕⊕〇 MODERATE | 3,051 /4,462 | 2,457 /3,713 | **RR 1.024** (0.940 to 1.116) |
| Incidence of TRAE | 6,395 (9 RCTs) | Not serious | Serious^1^ | Not serious | Not serious | N/A^2^ | ⊕⊕⊕〇 MODERATE | 3,124 /3,464 | 2,747 /2,931 | **RR 0.968** (0.936 to 1.001) |
| Incidence of TRAE grade 3-5 | 6,999 (10 RCTs) | Not serious | Serious^1^ | Not serious | Not serious | Not serious | ⊕⊕⊕〇 MODERATE | 1,980 /3,871 | 1,674 /3,128 | **RR 0.869** (0.738 to 1.023) |
| Incidence of IRAE | 5,639 (7 RCTs) | Not serious | Serious^1^ | Not serious | Serious^3^ | N/A^2^ | ⊕⊕〇〇 LOW | 1,499 /3,085 | 624 /2,554 | **RR 3.093** (1.993 to 4.798) |
| Incidence of immune-related TRAE | 1,960 (4 RCTs) | Not serious | Serious^1^ | Not serious | Serious^3^ | N/A^2^ | ⊕⊕〇〇 LOW | 275 /1,073 | 171 /887 | **RR 1.725** (0.882 to 3.373) |
| Incidence of TRAE-related discontinuation | 7,000 (10 RCTs) | Not serious | Serious^1^ | Not serious | Serious^3^ | Not serious | ⊕⊕〇〇 LOW | 589 /3,872 | 217 /3,128 | **RR 1.711** (1.224 to 2.390) |
| Improvement of QOL score from baseline | 3,590 (6 RCTs) | Not serious | Not serious | Not serious | Serious^3^ | N/A^2^ | ⊕⊕⊕〇 MODERATE | - | - | **SMD 0.048** (-0.106 to 0.202) |
| The proportion of patient with QOL improvement | 1,403 (4 RCTs) | Not serious | Not serious | Not serious | Serious^3^ | N/A^2^ | ⊕⊕⊕〇 MODERATE | 248 /804 | 176 /599 | **RR 1.106** (0.961 to 1.272) |
| Time to definitive QOL deterioration | 2,052 (3 RCTs) | Not serious | Serious^1^ | Not serious | Not serious | N/A^2^ | ⊕⊕⊕〇 MODERATE | - | - | **HR 0.508** (0.461 to 0.560) |

Abbreviations: **RCTs**: Randomized controlled trials; **CI**: Confidence interval; **HR**: Hazard ratio; **RR**: Risk ratio; **N/A**: Not applicable; **AE**: Adverse events of any causes; **TRAE**: Treatment-related adeverse events; **IRAE**: Immune-related adeverse events; **QOL**: Quality of life; **SMD**: Standardized mean differences

^1^ There was substantial-to-high heterogeneity among included studies.

^2^ Publication bias could not be determined as the number of studies was less than 10.

^3^ Wide confidence intervals in most of included studies.
