# Supplementary material for: Efficacy, safety, and patient-reported outcome of immune checkpoint inhibitor in gynecologic cancers: A systematic review and meta-analysis of randomized controlled trials
Source: PLoS One. 2024 Aug 12;19(8):e0307800. doi: 10.1371/journal.pone.0307800 (PMC11318932; doi:10.1371/journal.pone.0307800)
Supplement: S1 Fig — The diagram provided a summary of the search strategy and selection process used to include eligible articles for this meta-analysis. (DOCX) [file pone.0307800.s003.docx]

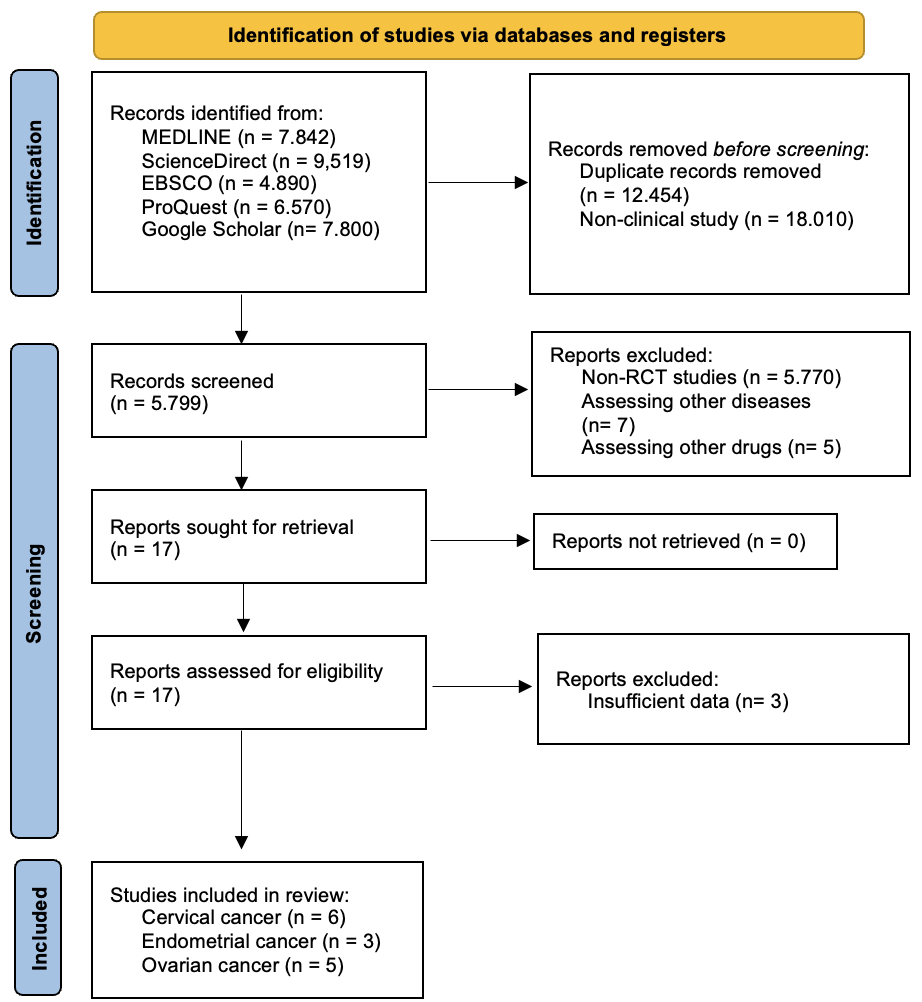


**Figure S1**. PRISMA Flowchart. The diagram provided a summary of the search strategy and selection process used to include eligible articles for this meta-analysis.
