# Supplementary figures and images for: Efficacy, safety, and patient-reported outcome of immune checkpoint inhibitor in gynecologic cancers: A systematic review and meta-analysis of randomized controlled trials
Source: PLoS One. 2024 Aug 12;19(8):e0307800. doi: 10.1371/journal.pone.0307800 (PMC11318932; doi:10.1371/journal.pone.0307800)

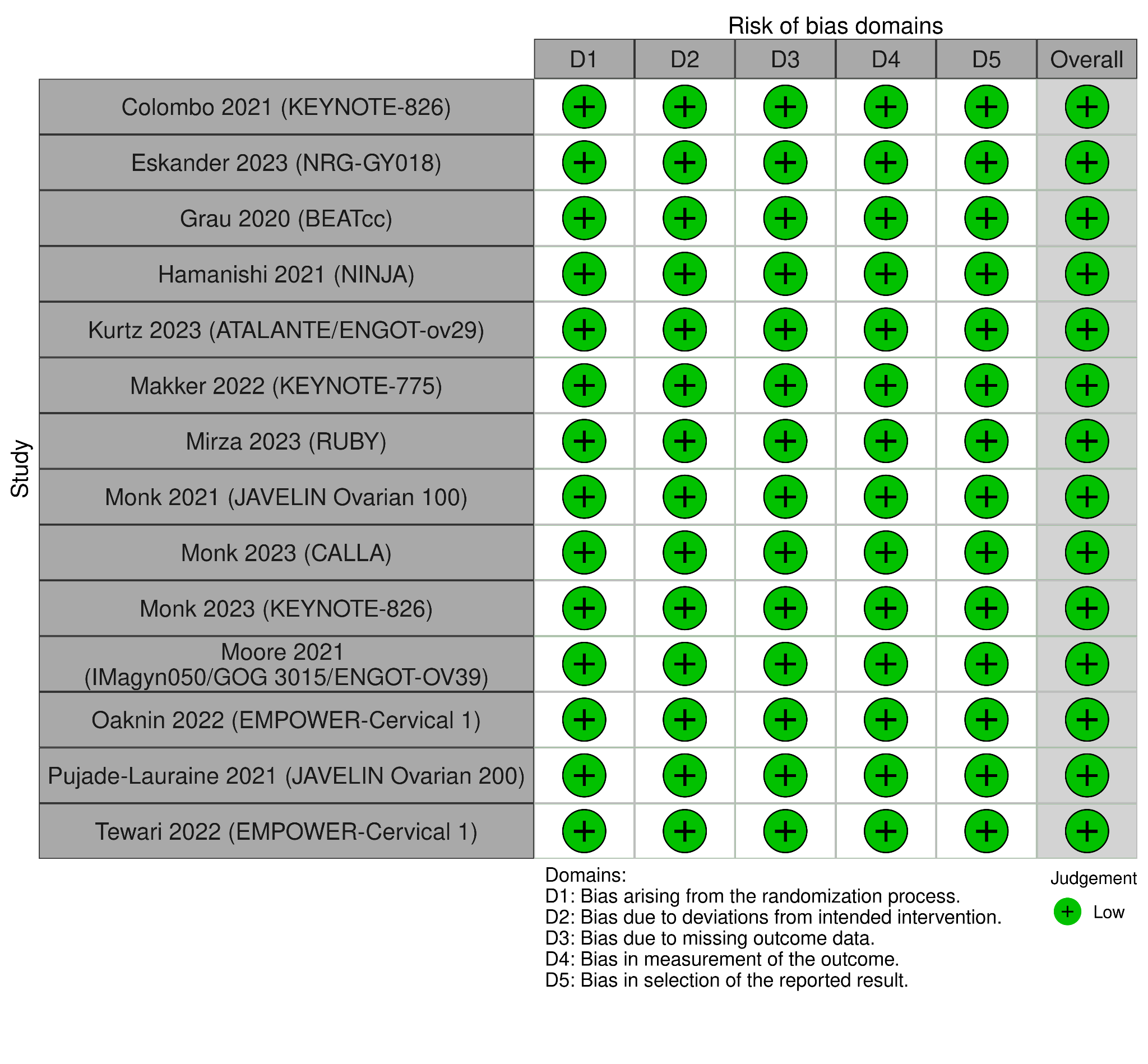


**Figure S2.** Quality Assessment. Cochrane risk of bias tool (ROB-2) was used to evaluate the risk of bias.

Supplement: S2 Fig — Cochrane risk of bias tool (ROB-2) was used to evaluate the risk of bias. (DOCX) [file pone.0307800.s004.docx]
