# Supplementary material for: Efficacy, safety, and patient-reported outcome of immune checkpoint inhibitor in gynecologic cancers: A systematic review and meta-analysis of randomized controlled trials
Source: PLoS One. 2024 Aug 12;19(8):e0307800. doi: 10.1371/journal.pone.0307800 (PMC11318932; doi:10.1371/journal.pone.0307800)
Supplement: S3 Fig — (Left) Forest plot. The horizontal line indicates 95% CI of a study. The square represents the result of each individual study. The size of the square varies according to the weight of a particular study. The diamond at the bottom of the plot represents the pooled analysis of all included studies. The outer edges of the diamond indicates the CIs. CI, confidence interval. (Right) Funnel plot. Asymmetrical plot indicated that publication bias was present. (DOCX) [file pone.0307800.s005.docx]

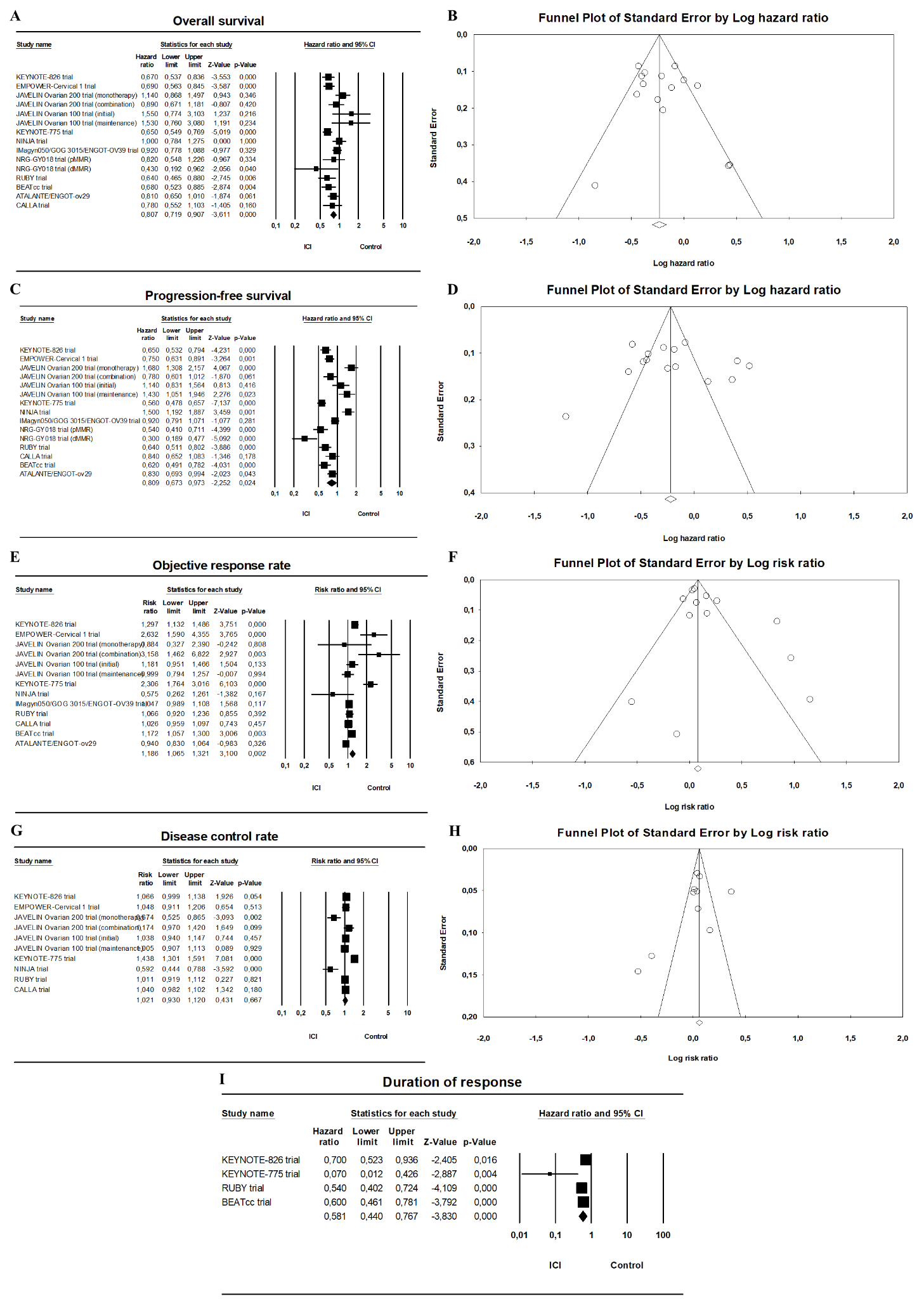


**Figure S3.** Overall survival, progression-free survival, objective response rate, disease control rate, and duration of response of ICI for gynecologic cancer. (Left) Forest plot. The horizontal line indicates 95% CI of a study. The square represents the result of each individual study. The size of the square varies according to the weight of a particular study. The diamond at the bottom of the plot represents the pooled analysis of all included studies. The outer edges of the diamond indicates the CIs. CI, confidence interval. (Right) Funnel plot. Asymmetrical plot indicated that publication bias was present.
